# Study of sRAGE, HMGB1, AGE, and S100A8/A9 Concentrations in Plasma and in Serum-Extracted Extracellular Vesicles of Pregnant Women With Preterm Premature Rupture of Membranes

**DOI:** 10.3389/fphys.2020.00609

**Published:** 2020-06-23

**Authors:** Damien Bouvier, Yves Giguère, Loïc Blanchon, Emmanuel Bujold, Bruno Pereira, Nathalie Bernard, Denis Gallot, Vincent Sapin, Jean-Claude Forest

**Affiliations:** ^1^Biochemistry and Molecular Genetic Department, Centre Hospitalier Universitaire (CHU) Clermont-Ferrand, Clermont-Ferrand, France; ^2^Faculty of Medicine, CNRS 6293, INSERM 1103, GReD, Université Clermont Auvergne, Clermont-Ferrand, France; ^3^Centre de Recherche du Centre Hospitalier Universitaire (CHU) de Québec-Université Laval, Québec City, QC, Canada; ^4^Department of Molecular Biology, Medical Biochemistry and Pathology, Faculty of Medicine, Université Laval, Québec City, QC, Canada; ^5^Department of Obstetrics and Gynecology, Faculty of Medicine, Université Laval, Québec City, QC, Canada; ^6^Biostatistics Unit Direction de la Recherche Clinique et des Innovations (DRCI), Centre Hospitalier Universitaire (CHU) Clermont-Ferrand, Clermont-Ferrand, France; ^7^Department of Obstetrics and Gynecology, Centre Hospitalier Universitaire (CHU) Clermont-Ferrand, Clermont-Ferrand, France

**Keywords:** preterm premature rupture of membranes, extracellular vesicles, soluble receptor for advanced glycation end products, advanced glycation end products, high-mobility group box 1, S100A8/A9

## Abstract

Preterm premature rupture of membranes (PPROM), defined as rupture of fetal membranes prior to 37 weeks of gestation, complicates approximately 2–4% of pregnancies and is responsible for 40% of all spontaneous preterm births. PPROM arises from complex pathophysiological pathways with a key actor: inflammation. Sterile inflammation is a feature of senescence-associated fetal membrane maturity. During specific steps of sterile inflammation, cells also release highly inflammatory damage-associated molecular pattern markers (DAMPs), such as high-mobility group box 1 (HMGB1) or S100A8/A9, known to link and activate the receptor for advanced glycation end products (RAGE). The objective of this study was to measure longitudinally during pregnancy concentrations of the soluble form of RAGE (sRAGE) and its main ligands (AGE, HMGB1, S100A8/A9) in blood specimens. We studied 246 pregnant women (82 with PPROM and 164 matched control pregnant women without complications) from a cohort of 7,866 pregnant women recruited in the first trimester and followed during pregnancy until delivery. sRAGE, AGE, HMGB1, and S100A8/A9 concentrations were measured in plasma and in serum-extracted extracellular vesicles from first trimester (T1), second trimester (T2), and delivery (D). In plasma, we observed, in both PPROM and control groups, (i) a significant increase of HMGB1 concentrations between T1 vs. T2, T1 vs. D, but not between T2 vs. D; (ii) a significant decrease of sRAGE concentrations between T1 and T2 and a significant increase between T2 and D; (iii) a significant decrease of AGE from T1 to D; (iv) no significant variation of S100A8/A9 between trimesters. In intergroup comparisons (PPROM vs. control group), there were no significant differences in time variation taking into account the matching effects. There was a correlation between plasma and serum-extracted extracellular vesicle concentrations of sRAGE, AGE, HMGB1, and S100A8/A9. Our results suggest that the rupture of fetal membranes (physiological or premature) is accompanied by a variation in plasma concentrations of sRAGE, HMGB1, and AGE. The study of RAGE and its main ligands in extracellular vesicles did not give additional insight into the pathophysiological process conducting to PPROM.

## Introduction

Preterm premature rupture of membranes (PPROM), defined as rupture of fetal membranes prior to 37 weeks of gestation, complicates approximately 2–4% of all pregnancies and is responsible for 40–50% of all preterm births (Naeye and Peters, 1980; Mercer et al., 2000). PPROM arises from complex, multifaceted pathophysiological pathways where the inflammation axis plays a major role (Menon and Richardson, 2017). Indeed, recent reports indicated that PPROM may be associated with sterile inflammation in the fetal membranes (Romero et al., 2015). In support of this hypothesis, it has been shown that histological chorioamnionitis in the presence of a negative amniotic fluid culture increases the risk of preterm birth (Park et al., 2017). Sterile inflammation is a feature of senescence-associated fetal membranes maturity and is characterized mostly by the presence of inflammatory biomarkers, growth factors, and matrix degrading enzymes (Coppe et al., 2008). During the specific steps of sterile inflammation, senescent, stressed, or necrotic cells release highly inflammatory damage-associated molecular pattern markers (DAMPs) (Menon and Richardson, 2017). High-mobility group box 1 (HMGB1) is one of the DAMPs that have been linked to parturition (Sheller-Miller et al., 2017; D’Angelo et al., 2018). In a mouse model, intra-amniotic administration of HMGB1 induces spontaneous preterm labor and birth (Gomez-Lopez et al., 2016). Moreover, it was observed that HMGB1 induces an inflammatory response, partially mediated by the inflammasome, in the fetal membranes (Plazyo et al., 2016). This intra-amniotic inflammasome activation was highlighted *in vivo* in human (Gomez-Lopez et al., 2019). HMGB1 is a known ligand of receptor for advanced glycation end products (RAGE), and the RAGE system is associated with pregnancy complications as preeclampsia or PPROM (Naruse et al., 2012; Rzepka et al., 2015). Moreover, AGEs could be implicated in PPROM with blood levels significantly higher in pregnant women complicated with PPROM (Kansu-Celik et al., 2019). Calprotectin (or S100A8/A9) is also a known ligand of RAGE (Pruenster et al., 2016) implicated in some pregnancy pathologies as preeclampsia (Pergialiotis et al., 2016).

Extracellular vesicles are a heterogeneous group of cell-derived membranous structures comprising exosomes (50–150 nm) and microvesicles (50–500 nm up to 1 μm), which originate from the endosomal system or which are shed from the plasma membrane, respectively (van Niel et al., 2018). The study of maternal plasma exosomes determines pathways associated with PPROM including non-specific inflammation or oxidative stress (Menon et al., 2019). The RAGE system (receptor and ligands) has not been specifically studied in maternal blood exosomes. However, some DAMPs, such as HMGB1 have been identified as present in oxidative-stressed amnion epithelial cell-derived exosomes (Sheller-Miller et al., 2017). Furthermore, *in vitro*, amnion epithelial cell exosomes lead to an increased inflammatory response in maternal uterine cells, suggesting that fetal cell exosomes may act as a signal to parturition in choriodecidua and migrate into the maternal circulation (Hadley et al., 2018). Combining maternal characteristics and environmental and clinical known risk factors (Bouvier et al., 2019) to candidate biomarkers may in the future result in proposing a clinically predictive model identifying asymptomatic women at higher risk of PPROM.

In this context, taking advantage of a large cohort of pregnant women recruited prospectively at the beginning of pregnancy, we investigated the changes in the concentrations of the soluble form of RAGE (sRAGE) and its main ligands (AGE, HMGB1, S100A8/A9) in plasma and in the serum-extracted extracellular vesicles from first trimester to delivery to better understand the potential role of the RAGE system in PPROM.

## Materials and Methods

### Study Design and Participants

This is a case/control study of sRAGE, HMGB1, AGE, and S100A8/A9 concentrations in plasma samples and serum-extracted exosomes of pregnant women with PPROM from an already constituted prospective biobank for which blood samples were collected [research program funded by the CIHR Institute of Human Development, Child and Youth Health Initiative (Grant Number: NRFHPG-)]. The biobank includes samples from 7,866 pregnant women recruited at the CHU de Québec-Université Laval between April 2005 and March 2010 and followed during pregnancy until delivery (Forest et al., 2014). Participants gave their informed written consent, and the study was approved by the Ethics Committee of the CHU de Québec [initial approval date: November 9, 2004, project 5-04-10-01 [95.05.17], SC12-01-159]. Cases were selected from all pregnant women with PPROM for whom three successive blood samples were collected and then frozen: one during the first trimester (T1), one during the second trimester (T2), and one at delivery (D). In the control group, we selected pregnant women (two control for one case) with delivery at term (after 37 weeks of gestation) and for whom three plasma and serum samples were collected and then frozen (T1, T2, and D). Women in the control group were matched with those in the case group on the following criteria: maternal age (±5 years), gestational age at T1 sample (±1 week), gestational age at T2 sample (±3 weeks), storage time at -80°C (±6 months). A total of 246 pregnant women (82 with PPROM and 164 matched control pregnant women) were selected.

### Serum Extracellular Vesicle Extraction

For total extracellular vesicles from 30 to 120 nm isolation from 738 serum samples (three samples T1, T2, and D for 246 women), we used a kit (ref 4478360) from Invitrogen^TM^ (Carlsbad, California, United States) using 450 μl of serum and following the manufacturer’s instructions. Then, for extraction of total proteins from extracellular vesicles, we used Invitrogen^TM^ kit (ref 4478545) following the manufacturer’s instructions. The assay of total proteins in the extracellular vesicle extracts was carried out using a Vista^®^ analyzer (Siemens, Munich, Germany). The assay of apolipoprotein B (Apo B) in 16 extracellular vesicle extracts (eight from the control group and eight from the PPROM group) was carried out using a Vista^®^ analyzer (Siemens, Munich, Germany).

### ELISA of Soluble Receptor for Advanced Glycation End Products, High-Mobility Group Box 1, Advanced Glycation End Products, and S100A8/A9

The concentrations of sRAGE, HMGB1, AGE, and S100A8/A9 in 738 plasma samples (three samples T1, T2, and D for 246 women) and in 738 serum-extracted extracellular vesicle samples (three samples T1, T2, and D for 246 women) was measured by the ELISA method using MyBioSource^®^ kits (San Diego, California, United States) following the manufacturer’s instructions (ref MBS2515963, MBS024146, MBS2000151, and MBS7606803, respectively). The concentrations of sRAGE, HMGB1, AGE, and S100A8/A9 of each serum extracellular vesicle sample were normalized against total protein concentrations.

### Statistics

Statistical analyses were performed using Stata software, Version 13 (StataCorp, College Station, Texas, United States). All tests were two-sided, with a Type I error set at 0.05. Continuous data were expressed as mean and standard deviation (SD) or median and interquartile range (IQR) according to statistical distribution. The assumption of normality was assessed by using the Shapiro–Wilk test. The comparisons between the PPROM and control groups, for non-repeated data, were performed using Student *t*-test or Mann–Whitney test when the assumptions of *t*-test were not met for continuous parameters. Chi-square test or, if applicable, Fisher’s exact test were applied for categorical variables. The relation between continuous variables (AGEs, sRAGE, HMGB1, S100A8/A9 concentrations) in serum-extracted extracellular vesicles and in plasma was analyzed estimating correlation coefficients, Pearson or Spearman according to the statistical distribution and applying a Sidak’s type I error correction to take into account multiple comparisons. These correlations’ results were illustrated with a color-coded heat map.

Random-effects models for repeated data were performed to compare the evolution of AGEs, sRAGE, HMGB1, and S100A8/A9 plasma concentrations and serum-extracted extracellular vesicle concentrations between groups (PPROM and controls). The following fixed effects were measured: time (T1, T2, D), group and *time* × *group* interaction, taking into account between- and within-participant variability (subject as random-effect). The normality of residuals from these models was studied using the Shapiro–Wilk test. When appropriate, a logarithmic transformation was proposed to achieve the normality of dependent outcome. A Sidak’s type I error correction was applied to perform multiple comparisons.

## Results

### Description of the Cohort

Of the 7,866 pregnant women recruited for the biobank, 189 women presented a PPROM (2.4%). Of these, 82 fulfilled the criteria of disposing of three blood samples. Therefore, a total of 246 pregnant women (82 with PPROM and 164 matched control pregnant women, 1:2 ratio) were selected. No significant differences (*p* = 0.7) were observed between the mean age of mothers in the control group (29.5 years, SD: 4.1) and the PPROM group (29.3 years, SD: 4.2) (Table 1). Some risk factors of PPROM were found significantly higher in the PPROM group as nulliparity, past history of PPROM, gestational diabetes mellitus, smoking during pregnancy (Table 1). A significant difference (*p* < 0.001) for the gestational age at delivery was expectedly observed between the PPROM group [36 weeks, interquartile range (IQR): 35.1–36.4] and the control group (38.7 weeks, IQR: 38.1–39.3) (Table 1).

**TABLE 1:** Characteristics of the case (PPROM) and control groups.

		PPROM group	Control group	p
n		82	164	/
Mean age of mothers (SD) in years		29.3 (4.2)	29.5 (4.1)	0.7
Nulliparity in%		58.5	43.3	0.02
Past history of PPROM in%		15.6	0	<0.001
Gestational diabetes mellitus in%		14.6	6.7	0.04
Smokers during pregnancy in%		21.3	11.7	0.04
Median	First trimester	15.1 (14–16.6)	15 (14–15.6)	0.37
gestational age	Second trimester	27.9 (26.1–28.5)	27.9 (26.2–28.4)	0.97
(IQR) in weeks	Delivery	36 (35.1–36.4)	38.7 (38.1–39.3)	<0.001

**IQR, interquartile range; PPROM, preterm premature rupture of membranes; SD, standard deviation.**

### Assays in Plasma

**FIGURE 1:**
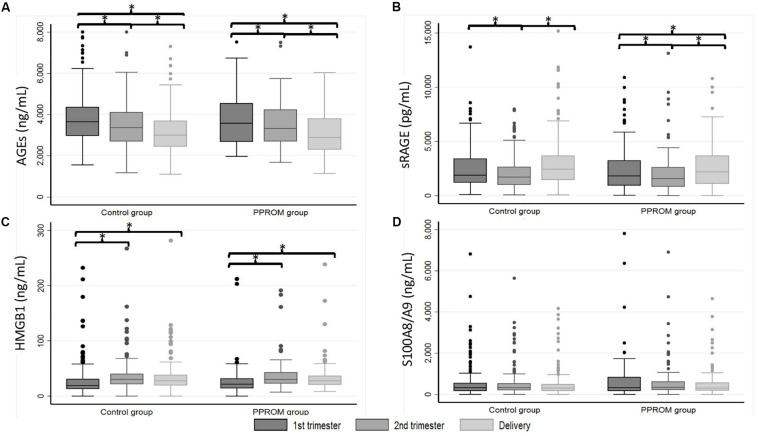
Box plot of AGEs **(A)**, sRAGE **(B)**, HMGB1 **(C)**, and S100A8/A9 **(D)** plasma median concentrations from 82 women in the PPROM group and 164 women in the control group at three points: first trimester, second trimester, and delivery. *p < 0.05 (random-effects for repeated measures). AGEs, advanced glycation end products; HMGB1, high-mobility group box 1; PPROM, preterm premature rupture of membranes; sRAGE, soluble receptor of advanced glycation end products.

A significant decrease of median concentrations of AGEs in both PPROM and control groups was observed between T1 and T2, T2 and D, and T1 and D (see p^1^ and p^2^ in Table 2 for the PPROM and control group, respectively; Figure 1A). These variations in concentrations observed during pregnancy were not significantly different between the PPROM and control groups (see p^3^ in Table 2). Similarly, for each sampling time (T1, T2, D), the medians of plasma concentrations are not significantly different between the PPROM and control groups (see p^4^ in Table 2).

**TABLE 2:** Median AGEs, sRAGE, HMGB1, and S100A8/A9 plasma concentrations from 82 women in the PPROM group and 164 women in the control group at three points: first trimester (T1), second trimester (T2), and delivery (D).

	PPROM group (*n* = 82)		Control group (*n* = 164)		p^3^	p^4^
	Median (IQR)	p^1^ T1 vs. T2 T2 vs. D D vs. T1	Median (IQR)	p^2^ T1 vs. T2 T2 vs. D D vs. T1		
**AGEs (ng/ml)**						
First trimester	3,569 (2,665–4,532)	0.04	3,651 (2,951–4,345)	<0.001	0.16	0.41
Second trimester	3,313 (2,676–4,232)	<0.001	3,353 (2,692–4,125)	<0.001	0.17	0.76
Delivery	2,885 (2,284–3,818)	<0.001	2,993 (2,439–3,694)	<0.001	0.97	0.43
**sRAGE (pg/ml)**						
First trimester	1,819 (915–3,234)	0.001	1,873 (1,170–3,402)	<0.001	0.33	0.55
Second trimester	1,556 (808–2,625)	<0.001	1,719 (971–2,670)	<0.001	0.65	0.30
Delivery	2,178 (1,092–3,721)	0.06	2,418 (1,432–3,665)	<0.001	0.15	0.21
**HMGB1 (ng/ml)**						
First trimester	22.3 (14.1–33.9)	<0.001	19.1 (13–31.3)	<0.001	0.65	0.32
Second trimester	30.4 (22.6–44.3)	0.24	30.4 (21.5–40.7)	0.09	0.86	0.29
Delivery	27.8 (20.5–37.7)	<0.001	27.7 (19–38.9)	<0.001	0.78	0.26
**S100A8/A9 (ng/ml)**						
First trimester	332 (157–865)	0.77	344 (168–692)	0.61	0.98	0.68
Second trimester	344 (216–644)	0.14	325 (204-678)	0.55	0.33	0.64
Delivery	297 (163–568)	0.24	316 (157–673)	0.93	0.31	0.23

**p^1^ and p^2^: intragroup comparison between T1, T2, and D in the PPROM group (p^1^) and in the control group (p^2^) (random-effects models for repeated measures). p^3^: intergroup comparison: interaction time × group, taking into account the matching effect (random-effects models for repeated measures). p^4^: comparison (of medians) between the PPROM group and the control group, taking into account the matching effect. AGEs, advanced glycation end products; HMGB1, high-mobility group box 1; IQR, interquartile range; PPROM, preterm premature rupture of membranes; sRAGE, soluble receptor of advanced glycation end products.**

For sRAGE in both PPROM and control groups, a significant decrease of median concentration between T1 and T2 and then a significant increase between T2 and D were observed (see p^1^ and p^2^ in Table 2 for the PPROM and control group, respectively; Figure 1B). At delivery, the sRAGE concentration was significantly higher than at T1 in the control group (*p* < 0.001) but not in PPROM (*p* = 0.06). These variations in concentrations observed during pregnancy were not significantly different between the PPROM and control groups (see p^3^ in Table 2). Similarly, for each sampling time (T1, T2, D), the medians of plasma concentrations were not significantly different between the PPROM and control groups (see p^4^ in Table 2).

For HMGB1 in both PPROM and control groups, a significant increase of median concentration was observed between T1 and T2 (and also T1 and D) followed by a stagnation between T2 and D (see p^1^ and p^2^ in Table 2 for the PPROM and control groups, respectively; Figure 1C). These variations in concentrations observed during pregnancy were not significantly different between the PPROM and control groups (see p^3^ in Table 2). Similarly, for each sampling time (T1, T2, D), the medians of plasma concentrations were not significantly different between the PPROM and control groups (see p^4^ in Table 2).

For S100A8/A9, in both PPROM and control groups, no significant variations of median concentrations were observed between T1, T2, and D (see p^1^ and p^2^ in Table 2 for the PPROM and control groups, respectively, see p^3^ in Table 2 and Figure 1D). Similarly, for each sampling time (T1, T2, D), the medians of plasma concentrations were not significantly different between the PPROM and control groups (see p^4^ in Table 2).

### Assays in Serum-Extracted Extracellular Vesicles

**FIGURE 2:**
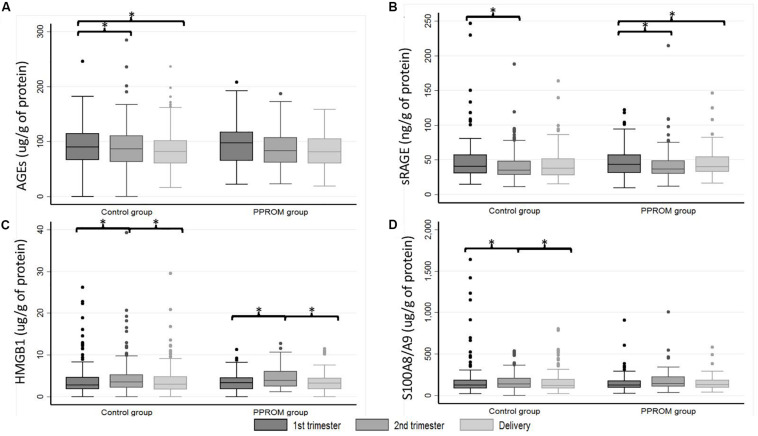
Box plot of AGEs **(A)**, sRAGE **(B)**, HMGB1 **(C)**, and S100A8/A9 **(D)** serum-extracted extracellular vesicle median concentrations from 82 women in the PPROM group and 164 women in the control group at three points: first trimester, second trimester, and delivery. *p < 0.05 (random-effects for repeated measures). AGEs, advanced glycation end products; HMGB1, high-mobility group box 1; PPROM, preterm premature rupture of membranes; sRAGE, soluble receptor of advanced glycation end products.

**FIGURE 3:**
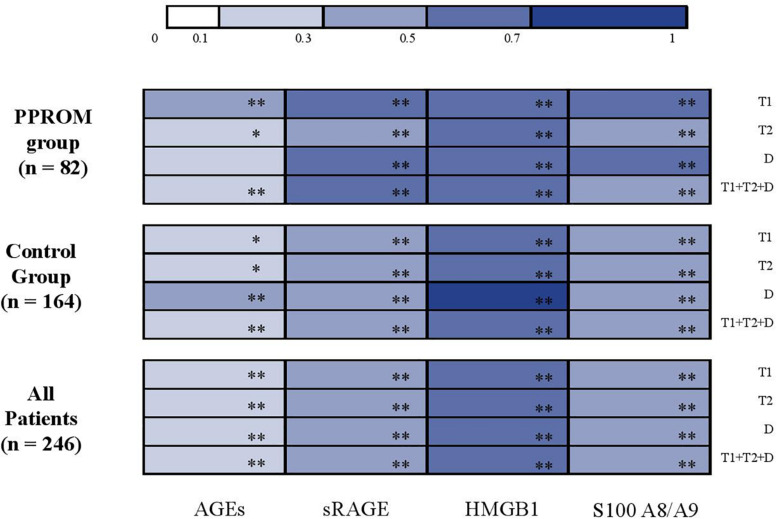
Correlation analysis between AGEs, sRAGE, HMGB1, and S100A8/A9 concentrations in serum-extracted extracellular vesicles and in plasma (color-coded heat map plot). AGEs, advanced glycation end products; D, delivery; HMGB1, high-mobility group box 1; PPROM, preterm premature rupture of membranes; sRAGE, soluble receptor of advanced glycation end products; T1, first trimester; T2, second trimester. *p < 0.05; **p < 0.001.

Similar results as those obtained in plasma are presented in Table 3 were observed. The variations of concentration during pregnancy were similar as in plasma (see p^1^ and p^2^ in Table 3 for the PPROM and control groups, respectively; Figure 2). For all four markers (AGEs, sRAGE, HMGB1, and S100A8/A9) measured in serum-extracted extracellular vesicles, the variations in concentration observed during pregnancy were not significantly different between the PPROM group and the control group (see p^3^ in Table 3). Similarly, for each sampling time (T1, T2, D), the medians of serum-extracted extracellular vesicles concentrations were not significantly different between the two groups (see p^4^ in Table 3). Moreover, AGEs, sRAGE, HMGB1, and S100A8/A9 concentrations were significantly correlated between the plasma and the serum-extracted extracellular vesicles for both the PPROM group (expect for AGEs at delivery) and the control group (Figure 3).

**TABLE 3:** Median AGEs, sRAGE, HMGB1, and S100A8/A9 serum-extracted extracellular vesicle concentrations from 82 women in the PPROM group and 164 women in the control group at three points: first trimester (T1), second trimester (T2), and delivery (D).

	PPROM group (*n* = 82)		Control group (*n* = 164)		p^3^	p^4^
	Median (IQR)	p^1^ T1 vs. T2 T2 vs. D D vs. T1	Median (IQR)	p^2^ T1 vs. T2 T2 vs. D D vs. T1		
**AGEs (μ g/g of protein)**						
First trimester	97.4 (64.7–117.5)	0.01	90 (66.3–115.1)	0.20	0.53	0.40
Second trimester	83.3 (61.2–107)	0.77	86.3 (63–110.3)	0.97	0.85	0.89
Delivery	81.4 (59.4–105.2)	0.01	81.6 (60.1–101.6)	0.22	0.41	0.91
**sRAGE (ng/g of protein)**						
First trimester	43.3 (30.6–57.3)	0.004	40.6 (30.3–57.6)	<0.001	0.42	0.92
Second trimester	36.7 (29.6–48.8)	0.07	34.7 (27.9–48.4)	0.06	0.73	0.43
Delivery	40.1 (32.3–54.2)	0.29	37.9 (27.4–51.7)	0.001	0.25	0.27
**HMGB1 (μ g/g of protein)**						
First trimester	3.4 (1.8–4.7)	<0.001	2.8 (1.8–4.6)	0.001	0.23	0.59
Second trimester	4.1 (2.4–6.2)	<0.001	3.5 (2.1–5.2)	<0.001	0.21	0.11
Delivery	3.2 (1.8–4.4)	0.63	2.9 (1.7–4.7)	0.64	0.95	0.66
**S100A8/A9 (μ g/g of protein)**						
First trimester	125 (93–174)	0.008	123 (86–186)	0.84	0.11	0.80
Second trimester	143 (107–226)	0.001	140 (95–205)	0.25	0.11	0.16
Delivery	127 (91–183)	0.39	119 (85–191)	0.34	0.99	0.89

**p^1^ and p^2^: intragroup comparison between T1, T2, and D in the PPROM group (p^1^) and in the control group (p^2^). p^3^: intergroup comparison: interaction time × group, taking into account the matching effect (random-effects models for repeated measures). p^4^: comparison (of medians) between the PPROM group and the control group, taking into account the matching effect (random-effects models for repeated measures). AGEs, advanced glycation end products; HMGB1, high-mobility group box 1; IQR, interquartile range; PPROM, preterm premature rupture of membranes; sRAGE, soluble receptor of advanced glycation end products.**

The assay of Apo B in 16 extracellular vesicles extracts (eight from the control group and eight from the PPROM group) was carried out. The results were found to be below or at the lower limit of linearity (<0.26 g/L).

## Discussion

We studied for the first time, on a large number of pregnant women (with and without PPROM), the kinetics from the first trimester to delivery of the plasma concentrations (and serum-extracted exosomes) of four major actors of the RAGE system: sRAGE and three of its ligands, HMGB1, AGEs, and S100A8/9. Among the 189 women with PPROM (2.4% of the cohort), 82 had three blood samples during their pregnancies and were matched to normal controls in a ratio of 1–2. Despite the potential bias linked to the matching criteria, we identified the clinical known risk factors for PPROM as described in a previous study (Bouvier et al., 2019).

There were no significant differences in the serum concentration of total circulating extracellular vesicles between pregnant women with and without PPROM. However, it was proposed that the study of maternal plasma-extracted exosomes could determine pathways associated with PPROM including non-specific inflammation or oxidative stress (Menon et al., 2019). The specific study of biomarkers of the RAGE system in serum-extracted extracellular vesicles of pregnant women was relevant to identify an earlier signal of suffering from the fetal membranes. In this study, we observed that this is not the case and that the concentrations are strongly correlated to those of “total serum,” for the studied actors of RAGE signaling, in contrast to the cellular networks in the work of Menon’s team. Flow cytometry could have been used to determine whether RAGE or studied DAMPs are present on the surface of or inside the extracellular vesicles, but this was not available in our current setting. Another limitation of the study is the absence of characterization of the extracellular vesicles extracted from the serum (van Niel et al., 2018) and of the study of the cellular or tissue origin of these vesicles. Measurement of Apo B below or at the lower limit of linearity (<0.26 g/L) confirmed the absence of contamination of the preparations of extracellular vesicles.

So far, studies on blood levels of sRAGE as PPROM or prematurity risk factor show discordant results (Hajek et al., 2008; Germanova et al., 2010; Bastek et al., 2012; Rzepka et al., 2016). In our study, no differences in plasma sRAGE concentrations were observed between the control and PPROM groups, as already described (Hajek et al., 2008; Rzepka et al., 2016). Indeed, Rzepka et al. (2016) found the same results and found the endogenous secretory RAGE (esRAGE) more interesting as a potential biomarker of PPROM. Bastek et al. (2012) found lower serum concentrations of sRAGE in women who gave birth prematurely including spontaneous premature labor with intact membranes and thus confirmed results of a smaller study (Germanova et al., 2010). Conversely, Hajek et al. (2008) found, in a pilot study, higher values of serum concentrations of sRAGE in women who gave birth prematurely. In our study, an interesting kinetics is observed with a decrease of sRAGE plasma concentration between T1 and T2, and then an increase between T2 and D. Germanova et al. (2010) described exactly the opposite but on a smaller cohort of 79 women with only 25 measurements per trimester. These different conclusions could be due to the presence of different size of cohorts, type of circulating RAGE, and various assays used to measure sRAGE.

Concerning the RAGE ligands in plasma, we observed no differences in S100A8/19 concentrations between the PPROM group and the control group and no variations during pregnancy. Also, we found no differences in plasma AGE concentrations between the PPROM and control groups. These results are in contradiction with those of a recent study on a small cohort of 46 pregnant women (nine with PPROM and 37 without PPROM) where the blood concentration of AGEs at T1 was higher in women with PPROM (Kansu-Celik et al., 2019). Noteworthy, a decrease in the serum concentration of AGEs during pregnancy was also observed in a previous study where the kinetics were studied between the second trimester and delivery (Quintanilla-Garcia et al., 2018). In our study, no differences in plasma HMGB1 concentrations were observed between the PPROM and control groups. However, variations in plasma HMGB1 concentrations during pregnancy were observed with a significant increase between T1 and T2 and then stagnation between T2 and D in both PPROM and control groups. It was previously observed that senescent fetal membranes contribute to sterile inflammation by generation of DAMPs, like HMGB1 (Menon et al., 2016). Also, an increase in the expression of HMGB1, with activation of the RAGE pathway, in the placenta of women with PPROM has been demonstrated (Yan et al., 2018). Our results show the same kinetics in women with PPROM and in control women. HMGB1 has a physiological implication and can be at the “frontiers in physiology.” Based on our blood results, the mechanistic link between the increases in plasma concentrations of HMGB1 and sRAGE and the decrease in the plasma concentration of AGEs should be further investigated. It has been reported that the sRAGE–AGE complex becomes degraded in the spleen or liver (Ramasamy et al., 2008).

## Conclusion

In conclusion, our results suggest that the rupture of fetal membranes (physiological or premature) may be related to RAGE activation possibly by the ligand HMGB1. If a more important production of HMGB1 occurring during pregnancy with PPROM could not be detected directly by a higher concentration of HGMB1 in PPROM vs. control group, an indirect proof by the different kinetics of sRAGE between first trimester and delivery could be proposed.

## Data Availability Statement

All datasets generated for this study are included in the article/supplementary material.

## Ethics Statement

The studies involving human participants were reviewed and approved by the Ethics Committee of the CHU de Québec. The patients/participants provided their written informed consent to participate in this study.

## Author Contributions

DB analyzed and interpreted the data and wrote the initial version of the manuscript. J-CF and YG were in charge of the research program on pregnancy complications, designed the study, and assisted with the interpretation of the data and writing of the manuscript. NB and EB supervised the trial and data collection. DB and NB carried out serum exosome extractions and all assays. BP provided statistical advice for the study design and analyzed the data. VS, LB, and DG experts in PPROM, reviewed the manuscript, assisted with the interpretation of the data. All authors substantially contributed to its revision.

## Conflict of Interest

The authors declare that the research was conducted in the absence of any commercial or financial relationships that could be construed as a potential conflict of interest.
